# A cluster-randomised controlled feasibility trial evaluating the Cognitive Occupation-Based programme for people with Multiple Sclerosis (COB-MS)

**DOI:** 10.1007/s10072-024-07757-5

**Published:** 2024-09-24

**Authors:** Sinéad M. Hynes, Christopher P. Dwyer, Alberto Alvarez-Iglesias, Fionnuala Rogers, Robert A. Joyce, Megan H. Oglesby, Anusha Moses, Eimear Bane, Timothy J. Counihan, Beatrice Charamba, COB-MS PPI Advisory Panel 1

**Affiliations:** 1 Discipline of Occupational Therapy, School of Health Sciences, College of Medicine, Nursing and Health Sciences, University of Galway, Galway, Ireland; 2 HEA Performance & Department of Teacher Education, Technological University of the Shannon, Athlone, Ireland; 3 Health Research Board Clinical Research Facility, University of Galway and School of Medicine, University of Galway, Galway, Ireland; 4 Cardiff University Brain Research Imaging Centre (CUBRIC), Cardiff University, Cardiff, Wales UK; 5 Faculty of Science and Technology, University of Twente, Enschede, Netherlands; 6 School of Psychology, University of Galway, Galway, Ireland; 7 Department of Neurology, University Hospital Galway, Galway, Ireland; 8 Staburo GmbH, Aschauer Str. 26a, 81549 Munich, Bavaria Germany

**Keywords:** Multiple sclerosis, Cognitive rehabilitation, Occupational therapy, Feasibility, Cluster randomised controlled trial

## Abstract

**Introduction:**

There is a high prevalence of cognitive difficulties in MS, but despite this, there are few programmes targeting cognition that focus on the ability to function well in everyday life. The Cognitive Occupation-Based programme for people with Multiple Sclerosis (COB-MS), an occupation-focused cognitive intervention, was developed to address this. It addresses both the functional difficulties and the wide-ranging symptoms that present in MS.

**Objective:**

Here we report on the results of a cluster-randomised controlled feasibility trial (ISRCTN11462710; registered 4th September 2019) evaluating the COB-MS in terms of feasibility and initial efficacy as a cognitive intervention for people with MS.

**Method:**

The eight-session COB-MS intervention was delivered remotely by occupational therapists to participants with MS in the intervention group. Following the end of the trial the COB-MS was delivered to the wait-list control group. Data was collected from people with MS experiencing cognitive difficulties at baseline, post-intervention, 12-weeks, and 6-month follow-up. The primary outcome measure was the Goal Attainment Scaling at 12 weeks. Data was also collected in the domains of cognition, quality of life, and mood.

**Results:**

One hundred and eighteen people with MS and cognitive difficulties were randomised to either usual care (*n* = 60) or COB-MS intervention (*n* = 58). Ninety-four participants were retained at 6-month follow-up. The COB-MS was found to be feasible, including trial procedures and protocol. Data indicates that the COB-MS is accepted by participants and had positive impacts on daily life. Those allocated to the COB-MS group had a significant improvement in the primary outcome compared to the control condition. Progression criteria set for the feasibility trial have been met therefore further testing of the COB-MS at a definitive trial is supported by the results.

**Conclusion:**

The results provide a strong basis for a pathway to a future definitive trial of COB-MS, with respect to both feasibility and preliminary, clinical efficacy.

**Trial Registration:**

ISRCTN11462710 Date of registration: 4th September 2019.

**Supplementary Information:**

The online version contains supplementary material available at 10.1007/s10072-024-07757-5.

## Introduction

### Background

It is estimated that between 2.2 and 2.8 million people worldwide have multiple sclerosis (MS; [1, 2]. MS is a complex disease that is characterised by inflammatory demyelination and degeneration with resulting damage to the white and grey matter of the central nervous system [3, 4]. Cognitive difficulties are a prevalent, distressing and debilitating symptom of multiple sclerosis [5]. It is typically reported that up to 65% of people with MS experience a decline in their cognitive functioning [6–9], with memory, executive functions, processing speed and attention being the most affected areas [10].

Cognitive difficulties have significant impacts on quality of life, increase the likelihood of being unemployed, having depression, and having difficulty managing self-care and daily life activities [11, 12]. Few people with MS receive intervention for cognitive difficulties (e.g. [13, 14]) despite the debilitating impact that it can have. For example, people with MS who experience cognitive difficulties are 49% more likely to be unemployed than those not experiencing cognitive difficulties [15]. As well as an economic burden, cognitive symptoms in MS have been shown to be a major cause of disability and negatively impact quality of life [16].

Given the strain that many health-care services are under, particularly since the COVID-19 pandemic, having an accessible and low-cost intervention is a global priority, with tele/online interventions facilitating greater access to those underserved by more traditional healthcare models [17]. Although it is commonly occupational therapists who assess and treat cognitive dysfunction in MS in the UK [13] and Ireland [14] there are few, if any, cognitive interventions to alleviate the decline in cognition for people with MS that target meaningful activities of daily life (or occupations). The overall evidence for cognitive rehabilitation in MS is promising, with short-term results in subjective memory, quality of life, verbal memory, and information processing found [18]. There still exists an urgent need to develop this evidence-base to support people with MS to manage their cognitive difficulties in daily life.

The Cognitive Occupation-Based programme for people with Multiple Sclerosis (COB-MS; [19–21] has been developed to address the clinical gap that exists in cognitive care for MS. The COB-MS enables people with MS to identify, understand and learn new strategies to deal with their cognitive difficulties and is specific to the difficulties seen in MS. The programme was developed to provide holistic cognitive rehabilitation in MS and focuses on rehabilitation through an individualised cognitive intervention, measured by and taught through an occupational participation perspective- focused on engagement in everyday activities. The first step in evaluating the COB-MS is through feasibility testing, the results of which are presented here, following a published protocol and update in light of COVID-19 [21, 22].

### Aim and objectives

The aim of the current research is to evaluate the feasibility and preliminary efficacy of the COB-MS on cognitive and daily functioning for people with MS. Specifically, the objectives are to:

1. Assess the integrity of the protocol and field test the outcome measures and procedures used in the trial.
2. Determine the preliminary efficacy of COB-MS in comparison with treatment as usual.
3. Determine the acceptability of COB-MS and investigate the barriers and facilitators to using COB-MS.
4. Determine the appropriateness of progression to a definitive trial.

## Methods

For further methodological detail, see previous establishment of this feasibility trial’s protocol and its subsequent update [21, 22].

### Trial design

This study is reported in accordance with the CONSORT 2010 statement and the extensions for cluster trials ([23]; see Appendix 1). The current study is a single-blind, cluster-randomised controlled feasibility trial of COB-MS. The study used a treatment-as-usual (TAU), wait-list control group design and a pre-post study design with two additional follow-up testing times: 12 week and six-month follow-up (i.e. four data collection points). Follow-up data were collected to evaluate sustainability of intervention gains, if evident, as well as gathering data on retention over the entire duration of the trial.

People with MS were cluster-randomised to one of the two study arms. Specifically, they were assigned to occupational therapists, based on geographic location. Occupational therapists were randomly assigned using 1:1 allocation, via randomised block permutation (randomised blocks of four and six per block). Clustering was used as the intervention was planned to be delivered in in-person groups [21], but because of COVID-19 impacts the originally planned in-person intervention was delivered online. The randomisation was completed prior to the COVID-19 pandemic and the feasibility of this design was assessed through the trial [22].

### Participants

#### Setting

This was a community-based research study that was originally designed to run COB-MS groups at various locations across the Republic of Ireland. However, due to the arrival of COVID-19, the study protocol was amended and all assessments and interventions were subsequently administrated online, via *Zoom for Healthcare*. Despite this, the main study site remained the University of Galway and data were collected nationwide.

#### Recruitment and eligibility

Both occupational therapists and people with MS were recruited to the trial. Occupational therapists were recruited through a professional body email (Association of Occupational Therapists of Ireland) and through notification on the MS Ireland website, health professionals’ email list and the bi-annual MS Ireland research e-zine. Snowball sampling was also used, in which occupational therapists informed others potentially interested in the trial. Occupational therapists were eligible to participate if they were 1) CORU-registered and working as an occupational therapist in Ireland; 2) had experience working with people with MS; and 3) could commit to the requirements of the study, including online delivery of the COB-MS.

Initially, 50 occupational therapists expressed interest in participating as COB-MS session facilitators, of whom: three were not eligible and 26 declined participation, either explicitly or through null response. Notably, the primary reason for explicit decline was occupational therapists not obtaining permission from their service managers. Also, important to note, in light a six-month delay (resulting from the arrival of COVID-19), trial amendments (e.g. online delivery of the COB-MS) and both health-related and work-related concerns (Dwyer et al., 2023b), yielded an attrition of 13 occupational therapists. Thus, recruitment (using the same strategy as before) was again engaged and another 13 occupational therapists consented to take part. This left 21 occupational therapists (11 intervention arm; 10 wait-list control arm) who delivered the intervention and acted as clustering frame for allocation of people with MS.

People with MS were recruited through trial advertisement in relevant newsletters (e.g. monthly MS Ireland newsletter), on websites offering information and services to people living with MS (e.g. MS Ireland), social media, radio, local newspapers, and at public conferences in the Republic of Ireland. All individuals interested in participating self-selected through contacting the researchers by phone or email. Informed consent was obtained, and eligibility assessed prior to participation. Eligibility criteria were as follows (Table 1):

**Table 1:** Eligibility Criteria for participants with MS

Inclusion criteria. Participants:
• Were aged 18 years of age or older;
• Were fluent in written and spoken English;
• Had a diagnosis of multiple sclerosis (consistent with the McDonald Criteria for the Diagnosis of Multiple Sclerosis [29]);
• Had cognitive difficulties, as shown by a score of > 22 on the *Multiple Sclerosis Neuropsychological Screening Questionnaire* [30]
• Were clinically stable (i.e. not having an active relapse);
• Could provide informed consent;
• Had no neurologic history other than MS, including evidence of current dementia;
• Had no history of major depressive disorder, schizophrenia, or bipolar disorder I or II;
• Had no history of diagnosed substance use or dependence disorder;
• Were not currently undergoing any other form of cognitive rehabilitation;
• Were living in the community; • Had reliable internet connection to participate in online delivery of COB-MS
Exclusion criteria:
• Cognitive impairment that would affect reliable participation o All participants were assumed to have capacity to participate. If capacity or ability to participate was questioned, then potential participants were reviewed by research team members qualified to assess further (e.g. neurologist)

**Fig. 1:**
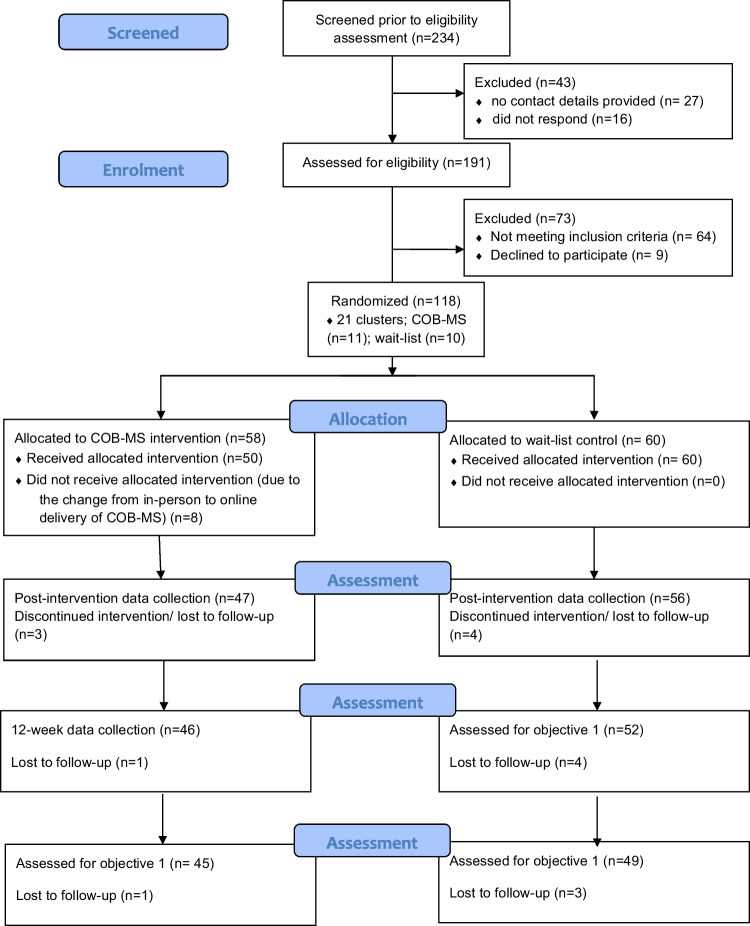
CONSORT Flow Diagram

In light of the aforementioned delay and protocol amendments resulting from COVID-19, seven previously consenting participants with MS declined progression onto the trial upon restart and one had become ineligible. Overall, 110 consenting PwMS participated (75f; 35 m), having completed baseline assessment and being randomly allocated to one of the two trial arms. See Fig. 1 for the Consort flow diagram of recruitment and retention in the trial.

### Interventions

#### Cognitive occupation-based programme for people with multiple sclerosis

The Template for Intervention Description and Replication (TIDieR; [24]) checklist was used here to describe the intervention (see Appendix 2).

#### Wait-list control treatment as usual

Participants randomised to the TAU, wait-list control arm of the study did not receive the COB-MS programme during the trial, but were provided access at the end of the data collection period, as delivered by the occupational therapist assigned to them upon randomised allocation. They received standard clinical care throughout the study’s life cycle, consistent with the aforementioned inclusion/exclusion criteria. Control participants were assessed at the same time points as the experimental arm.

Notably, the risk of contamination was low as cognitive rehabilitation is not standard care for patients with MS [14]. To reduce the chance of contamination, occupational therapists trained in the COB-MS were asked not to pass on their knowledge to non-COB-MS trained occupational therapists and this was part of the consent declaration. According to a recent national survey [14], ‘usual’ cognitive care for MS in Ireland typically conforms to the following:

- Occupational therapists are the health care professionals (HCP) most likely to assess and treat cognitive difficulties for people with MS.
- Only 34% of HCP who responded (*n* = 98) screen for cognitive difficulties in practice.
- 36% of HCPs provide information on cognition to patients.
- There appears to be very little consistency in cognitive assessment and treatment for people with MS.

The control group did not receive cognitive rehabilitation intervention during the trial period. Participants (across both arms) may have been taking medication that has an effect on cognition–e.g. benzodiazepine antispasmodics, anticholinergic agents. Participants continued with the pharmacological intervention.

### Outcomes

No changes were made to the outcomes after trial commencement. No adverse events were reported. All data were collected remotely. Self-report questionnaires were completed by participants either on paper (and posted back) or online through Microsoft Forms. Other outcomes were completed online with participants. The feasibility and equivalence of remote data collection were assessed and reported (see [25]).

#### Primary outcome

The Goal Attainment Scaling (GAS; [26]) was the primary outcome. The GAS allows participants to set meaningful goals relating to daily life which can be measured in a systematic way. GAS is responsive, shows reliability, validity and sensitivity [27], and has been used with people with MS (e.g. [28, 29]).

#### Secondary outcomes

Secondary outcome are listed here (for full description see protocol; [21]): Symbol Digit Modality Test (SDMT; [30]); California Verbal Learning Test II (CVLT-II; [31]); Trail Making Test (TMT; [32]); Brief Visuospatial Memory Test-Revised (BVMT-R; [33]); Everyday Memory Questionnaire Revised (EMQ-R; [34]); Generalised Self-Efficacy Scale (GSES; [35]); Modified Fatigue Impact Scale (MFIS; [36]); Multiple Sclerosis Quality of Life -54 (MSQoL-54; [37]); and General Health Questionnaire (GHQ-12; [38]).

#### Progression criteria

A traffic light system—green (go), amber (amend) and red (stop)—which allowed for modification was used [39], in consultation with the Trial Steering Committee (TSC). The key areas of risk were included in the criteria: trial recruitment, protocol adherence and outcomes.

Criteria in the acceptance checklist for clinical effectiveness pilot trials (ACCEPT; [40]) were used to evaluate progression – examining: 1) feasibility and appropriateness of the trial design; 2) feasibility and appropriateness of the mechanics, management and safety of interventions; and 3) acceptability and efficiency of implementing the research procedures.

### Sample size

A formal sample size calculation to evaluate the clinical effectiveness of COB-MS is not required give the focus on feasibility. A pragmatic approach was adopted that aimed at examining the rate of retention of participants during the intervention and follow-up periods. This was based on an average recruitment rate for funded trials from the National Institute for Health Research [41]. A 9% attrition rate was expected [42]. If randomised at the patient level a sample of 90 participants would allow for estimation of a retention rate of 91% with a 95% confidence interval (CI) of width equal to 13%. After allowing for clustering, assuming eight participants per cluster (occupational therapist) and an intracluster correlation coefficient (ICC) = 0.05, the sample size becomes 90 × [1 + (8–1) × 0.05] = 121. Thus, the number of occupational therapists needed is 121/8 = 15. A sample size of 15 × 8 = 120 participants with MS was calculated. Follow-up discussions with funders and trial steering committee recommended a final sample size of 100 participants as this was deemed large enough to provide information regarding the practicalities of a potential definitive randomised trial. This follows Consolidated Standards of Reporting Trials (CONSORT) guidelines for sample size calculation in feasibility studies. No interim analysis took place.

### Randomisation

All participants provided informed consent prior to randomisation. A web-based clinical trial randomisation service was used (Sealed Envelope), in which an unblinded member of the research team, independent of outcome data collection and analysis conducted the randomisation through Sealed Envelope’s platform.

Occupational therapists were registered to the trial and a unique identification code was assigned to them. Once the participants (with MS) were recruited to the trial and assigned to their corresponding occupational therapist, an unblinded member of the research team, independent of outcome data collection and analysis, generated the randomisation list at the cluster level, using randomly permuted blocks of size 4 and 6 in Sealed Envelope. The code used and the randomisation list was kept and securely stored by the independent researcher. All participants were informed of their allocation (with such implications explained) through both phone call and post/email. Participants’ details were then passed to their allocated occupational therapist to initiate contact and the intervention.

### Blinding

The study was single-blinded. The following people/groups were masked to participant allocation: all research staff collecting outcome measure data (not to include the qualitative data), statisticians and those involved in data analysis, and the TSC. It was not possible to mask the participants, nor the occupational therapists providing the intervention.

Participants were provided with written and video information on the importance of blinding and asked to conceal their group identity to research staff conducting outcome measure assessment. Blinded research staff did not have access to any data that might unblind them and were not present for team meetings where there was any risk to unblinding.

### Public and patient involvement (PPI)

This trial had a PPI member employed as a member of the research team for the entire duration of the trial. There were two PPI members on the TSC, and an external PPI consultation group was also convened. The PPI group contributed to decisions on key trial issues such as outcome measure selection, planning in light of COVID-19 pandemic, recruitment material, handbook design, qualitative evaluation and dissemination. PPI was critical to the success of the trial and was integrated through the entire trial life cycle. PPI processes were developed to evaluate the impact of the activities used [43].

### Statistical methods

The key outcomes in this study were the feasibility objectives set. The feasibility outcomes, recruitment rate, acceptability of COB-MS (from the perspective of participants with MS and occupational therapists), rate of unblinding, retention rate and randomisation methods are reported descriptively and narratively. Analysis took place once all data were collected.

Means and standard deviations (or medians and interquartile range [IQR] as appropriate) were used for continuous variables, with counts and percentages reported for categorical outcomes. The retention rate was estimated using a 95% CI. Estimates of the primary outcome variable (i.e. goal attainment scaling), at week 12, was used to inform sample size calculations of a future definitive trial. Data resulting from primary and secondary outcome measures was evaluated in terms of preliminary efficacy. Treatment effects were estimated using linear mixed models (including random intercepts at the occupational therapist level to account for the cluster structure) with the outcomes evaluated at week 12 and adjusted by baseline values. Trends in data over time are also presented to indicate the effect of the intervention over time. A qualitative evaluation of the acceptability of the COB-MS and related feasibility has been completed and reported [44].

## Results

### Participant demographics

Participant recruitment to the trial (to include informed consent) took place between 17/12/2019 and 10/03/2020. The final follow-up assessment was conducted on 1/10/2021. The trial concluded when all data were collected and the control group, subsequently, had received the COB-MS intervention (July 2022).

One-hundred and eighteen participants were randomised to intervention (*n* = 58) and control (*n* = 60) in 21 clusters (11 intervention; 10 control). There was a delay of six months between randomisation and intervention delivery due to COVID-19 (see 22). Participants were re-assessed at baseline because of the delay (see 25). The flow diagram of participants through the trial is in Fig. 1. The 110 remaining participants had a mean age of 48.22 years (SD9.98) and 67.9% were female. Relapsing remitting was the most common MS phenotype (69.1%) reported by participants, with secondary progressive (18.5%) the next most common, and primary progressive MS least commonly reported (8.6%) in participants (3.7% were unsure of MS phenotype). When asked if they considered cognition the primary symptom of their MS, 36.4% of participants said yes. Baseline data can be seen in Table 2.

**Table 2:** Participant Baseline Characteristics

		Overall [*N* = 110]	COB-MS [*N* = 50]	Wait-list Control [*N* = 60]
Demographics				
Age (years) [Mean (SD)]		48.2 (10) [N = 107]	49.6 (10.2) [N = 48]	47.9 (9.6) [N = 59]
Sex, % (*n*)	Female	67.3% (74)	66% (33)	68.3% (41)
	Male	32.7% (36)	34% (17)	31.7% (19)
Type of work, % (*n*)	Disabled	37% (30/81)	39.3% (11/28)	35.8% (19/53)
	Full Time	22.2% (18/81)	21.4% (6/28)	22.6% (12/53)
	Part Time	16% (13/81)	10.7% (3/28)	18.9% (10/53)
	Home Parent	13.6% (11/81)	10.7% (3/28)	15.1% (8/53)
	Retired	11.1% (9/81)	17.9% (5/28)	7.5% (4/53)
Marital status, % (*n*)	Married	63.4% (52/82)	57.1% (16/28)	66.7% (36/54)
	Single	13.4% (11/82)	10.7% (3/28)	14.8% (8/54)
	In a relationship	12.2% (10/82)	10.7% (3/28)	13% (7/54)
	Divorced	11% (9/82)	21.4% (6/28)	5.6% (3/54)
Multiple Sclerosis Phenotype				
Phenotype, % (*n*)	Relapsing–remitting	69.1% (56/81)	75% (21/28)	66% (35/53)
	Secondary Progressive	16% (13/81)	21.4% (6/28)	13.2% (7/53)
	Primary Progressive	8.6% (7/81)	3.6% (1/28)	11.3% (6/53)
	Not sure	3.7% (3/81)	0%	5.7% (3/53)
	Progressive Relapsing	2.5% (2/81)	0%	3.8% (2/53)
Multiple Sclerosis Symptoms				
Symptoms, % (*n*)	Cognition problems	100% (81/81)	100% (28/28)	100% (53/53)
	Fatigue	70.4% (57/81)	78.6% (22/28)	66% (35/53)
	Dizziness and Vertigo	37% (30/81)	42.9% (12/28)	34% (18/53)
	Numbness or Tingling	35.8% (29/81)	42.9% (12/28)	32.1% (17/53)
	Bladder Problems	33.3% (27/81)	46.4% (13/28)	26.4% (14/53)
	Weakness	30.9% (25/81)	39.3% (11/28)	26.4% (14/53)
	Other	25.9% (21/81)	28.6% (8/28)	24.5% (13/53)
	Vision Problems	21% (17/81)	25% (7/28)	18.9% (10/53)
	Bowel Problems	21% (17/81)	28.6% (8/28)	17% (9/53)
	Pain & Itching	18.5% (15/81)	17.9% (5/28)	18.9% (10/53)
	Spasticity	16% (13/81)	17.9% (5/28)	15.1% (8/53)
	Sexual Problems	14.8% (12/81)	14.3% (4/28)	15.1% (8/53)
	Emotional Changes	14.8% (12/81)	10.7% (3/28)	17% (9/53)
	Swallowing Problems	9.9% (8/81)	7.1% (2/28)	11.3% (6/53)
	Hearing Loss	8.6% (7/81)	7.1% (2/28)	9.4% (5/53)
	Depression	6.2% (5/81)	7.1% (2/28)	5.7% (3/53)
	Speech Problems	4.9% (4/81)	7.1% (2/28)	3.8% (2/53)
	Tremor	3.7% (3/81)	3.6% (1/28)	3.8% (2/53)
	Seizures	3.7% (3/81)	3.6% (1/28)	3.8% (2/53)
Is Cognition a primary symptom?, % (*n*)		63.6% (49/77)	70.4% (19/27)	60% (30/50)
Past conditions				
Stroke, % (*n*)		0%	0%	0%
Traumatic Brain Injury, % (*n*)		1.2% (1/81)	3.6% (1/28)	0%
Other neurological conditions, % (*n*)		2.5% (2/81)	3.6% (1/28)	1.9% (1/53)
Any psychiatric disorder, % (*n*)		2.5% (2/81)	0%	3.8% (2/53)

#### Medications

Among the 110 participants, 92 (84%) were taking some type of medication [44 (88%) in the COB-MS group and 48 (80%) in the Wait-list Control group]. Out of the 92 (84%) subjects taking any medication 76 (83%) took Disease-Modifying medications at some point during the trial [36 (82%) in the COB-MS group and 40 (83%) in the Wait-list Control group], and 67 (73%) took Symptomatic medications at any time during the trial [23 (52%) in the COB-MS group and 44 (92%) in the Wait-list Control group]. The following table (Table 3) summarises the types of medications by treatment arm (counts refer to the number of participants who had the medication at any time point during the trial).

**Table 3:** Participant Medications

	Overall [*N* = 92]	COB-MS [*N* = 44]	Wait-list Control [*N* = 48]	*p* value
Disease-Modifying				
Glatiramir Acetate, % (*n*)	7.6% (7)	9.1% (4)	6.2% (3)	0.706
Interferon-Beta, % (*n*)	13% (12)	11.4% (5)	14.6% (7)	0.761
Fingolimod, % (*n*)	15.2% (14)	18.2% (8)	12.5% (6)	0.565
Dimethyl Fumarate, % (*n*)	18.5% (17)	13.6% (6)	22.9% (11)	0.292
Natalizumab, % (*n*)	9.8% (9)	11.4% (5)	8.3% (4)	0.732
Cladribine, % (*n*)	3.3% (3)	4.5% (2)	2.1% (1)	0.605
Alumtuzumab, % (*n*)	2.2% (2)		4.2% (2)	0.495
Rituximab, % (*n*)	12% (11)	6.8% (3)	16.7% (8)	0.203
Ocrelizumab, % (*n*)	10.9% (10)	11.4% (5)	10.4% (5)	> 0.999
Teriflunomide, % (*n*)	1.1% (1)		2.1% (1)	> 0.999
Symptomatic				
Muscle Relaxants, % (*n*)	26.1% (24)	20.5% (9)	31.2% (15)	0.342
Mobility, % (*n*)	18.5% (17)	13.6% (6)	22.9% (11)	0.292
Fatigue, % (*n*)	16.3% (15)	11.4% (5)	20.8% (10)	0.267
Neuropathic Pain, % (*n*)	31.5% (29)	22.7% (10)	39.6% (19)	0.116
Bladder, % (*n*)	14.1% (13)	13.6% (6)	14.6% (7)	> 0.999
Other, % (*n*)	45.7% (42)	29.5% (13)	60.4% (29)	0.004

### Feasibility

With respect to trial engagement, completion and retention, of the 50 participants allocated to the COB-MS programme (experimental condition), 47 completed the post-intervention assessment (i.e. 94%), with 54% of all those allocated attending all eight sessions, 80% attending at least seven and 86% at least six. With the exception of one individual who dropped out prior to the first session, there was an 87.76% completion rate of all sessions. With respect to the other two participants who dropped out, one did so having completed three sessions and the other only attended one session. Of the controls, only four of the 60 allocated did not complete post-intervention assessment. Overall, 93.6% of the 110 participants allocated (i.e. across groups) completed post-intervention assessment – indicating feasibility with respect to having a > 90% completion rate; 89.1% completed T3 assessment; and 85.5% completed T4 assessment.

Regarding fidelity, each occupational therapist in the intervention arm completed audio-recordings, of two randomly selected COB-MS sessions. A fidelity check assessment form was completed for each recording. The average length of each session was 69 min (S1: 59 m; S2: 76 m; S3:74 m; S4: 78 m; S5: 76 m; S6: 70; S7: 68 m; and S8: 49 m), all of which were in a timeframe acceptably consistent with that proposed in the protocol. The content covered by occupational therapists was fully in-line with the programme outline, consistent with the session reports and audio-recordings submitted, with 100% fidelity observed from audio-recordings. All occupational therapists passed all aspects of the fidelity check assessment criteria.

Overall, occupational therapists (through focus groups) and participants (interviews) found COB-MS to be acceptable with respect to feasibility and appropriateness (see 44 for full description). Recommendations for minor amendments were made- examples include guidance on group sizes, scheduling of sessions, layout of handbook (more graphics) and availability of more online material for sessions.

### Harms

There were no harms or unintended effects of the intervention or the control condition. No adverse events were reported by participants, research staff, or occupational therapists.

### Preliminary efficacy

#### Primary outcome

**Fig. 2:**
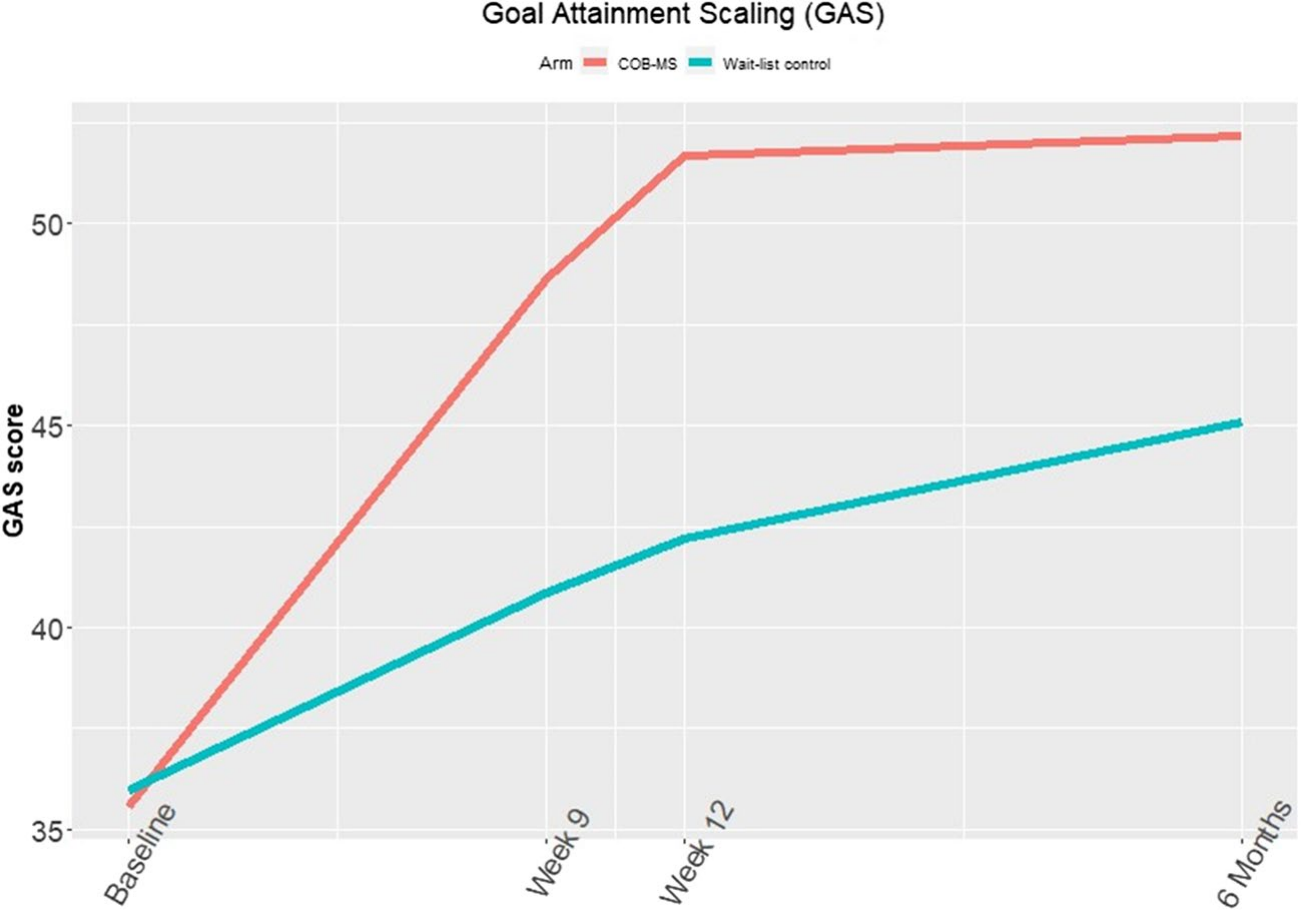
Mean GAS over time

Though the primary aim of the current research was feasibility testing, the preliminary efficacy of COB-MS was also evaluated. At week 12, the mean score of the primary outcome GAS in the COB-MS group was 51.7, 9.5 units higher than the mean score in the Wait-list, 42.2 (see Fig. 2). This difference was highly significant in a simple two-group comparison (*p*-value < 0.001). To account for the cluster structure, a linear mixed model was fitted with the GAS score as the outcome adjusted by the baseline scores and the grouping variable (including an OT level random intercept). The analysis showed that the OT-level variation was negligible and not large enough to warrant the inclusion of the random intercept. A simple linear regression model (including baseline as a covariate) was then adjusted, resulting in a mean difference between the groups of 9.5 (95% CI 5.6 to 13.4) at week 12 (see Table 4).

**Table 4:** Primary and secondary outcomes

Primary and secondary outcomes	Scale	COB-MS^a^	Wait-list Control^a^	Difference (95% CI)^b^
Goal Attainment Scaling (GAS)	Score	51.7 (11.1)	42.2 (7.8)	9.5 (95% CI 5.6 to 13.4)
Symbol Digit Modality Test (SDMT)	Correct Number of Substitutions	48 (15)	46.6 (14)	2.3 (95% CI -0.7 to 5.2)
Brief Visuospatial Memory Test-Revised (BVMT-R)	Total Recall, Median [IQR] (Raw Score)	23 (8.8)	20 (11)	3.6 (95% CI -0.5 to 7.7)
	Learning, Median [IQR] (Raw Score)	3 (2)	3 (1)	0.6 (95% CI -0.1 to 1.4)
	Delayed Recall, Median [IQR] (Raw Score)	9 (3)	7.5 (4)	1 (95% CI -0.2 to 2.1)
	Percent Retained, Median [IQR] (Raw Score)	100 (11)	100 (17.8)	3.6 (95% CI -6 to 13.2)
	Recognition Hits, Median [IQR] (Raw Score)	6 (1)	6 (1)	0 (95% CI -0.3 to 0.3)
	Recognition False Alarms, Median [IQR] (Raw Score)	0 (0)	0 (0)	0 (95% CI -0.4 to 0.3)
	Recognition Discrimination Index, Median [IQR] (Raw Score)	6 (1)	6 (1)	0 (95% CI -0.5 to 0.5)
	Recognition Response Bias, Median [IQR] (Raw Score)	0.5 (0)	0.5 (0.2)	0 (95% CI -0.1 to 0.1)
California Verbal Learning Test II (CVLT-II)	Total trials 1–5 (Raw Score)	54.1 (14)	52.5 (14.3)	1.6 (95% CI -2.9 to 6)
	Short-delay free recall (Raw Score)	11.3 (4.6)	10.8 (4.2)	0.2 (95% CI -1.2 to 1.5)
	Short-delay cued recall (Raw Score)	12.5 (3.7)	11.8 (3.4)	0.5 (95% CI -0.6 to 1.7)
	Long-delay free recall (Raw Score)	11.7 (4.5)	10.8 (4.1)	0.6 (95% CI -0.7 to 1.9)
	Long-delay cued recall (Raw Score)	12.3 (4.2)	11.7 (3.4)	0.4 (95% CI -0.8 to 1.6)
Trial Making Test	Trial A (seconds) Median [IQR]	0.019 (0.008)	0.02 (0.011)	0 (95% CI -0.006 to 0.006)
	Trial B (seconds) Median [IQR]	0.033 (0.021)	0.034 (0.02)	-0.001 (95% CI -0.017 to 0.014)
Everyday Memory Questionnaire—Relative (EMQ-R)	Total Score	14.5 (10)	17.1 (12.7)	-0.6 (95% CI -5.1 to 3.9)
General Self-Efficacy Scale (GSES)	Total Score	30.3 (5)	29.1 (5.2)	0.6 (95% CI -0.9 to 2.2)
Modified Fatigue Impact Scale (MFIS)	Physical subscale	20.1 (8)	23.3 (6.7)	-4 (95% CI -6.4 to -1.7)
	Cognitive subscale	20.1 (6.4)	23.8 (6.6)	-4.9 (95% CI -7.7 to -2.1)
	Psychosocial subscale	4.1 (2)	4.9 (2)	-1 (95% CI -1.7 to -0.3)
	Total Score	44.3 (14.1)	51.9 (12.7)	-10 (95% CI -15 to -4.9)
Multiple Sclerosis Quality of Life—54 (MSQOL-54)	Physical health composite	59.9 (20)	49.3 (16.3)	9 (95% CI 3.4 to 14.6)
	Mental health composite	71.9 (18.6)	60.5 (19.3)	8.6 (95% CI 1.1 to 16)
Short General Health Questionnaire (GHQ)	Total Score	9.7 (6.1)	13.8 (5.1)	-3.9 (95% CI -6.2 to -1.7)

^a^Summaries at week 12; ^b^Differences calculated using a linear mixed model (or a linear model where OT-level variation is negligible) with the outcome at week 12 as the response and adjusted by baseline values;

#### Secondary outcomes

Overall, across secondary outcomes there was an average trend towards better performance in the COB-MS group, though as can be seen below many of these differences did not reach statistical significance at week 12 (with the exception of the MFIS, MSQOL-54 and GHQ questionnaires). Results are presented in Table 4 and individual figures are available to download (Online Resource 1).

The analysis of trends over time for the primary and secondary outcomes was purely exploratory. We observed that, in general, differences detected after 12 weeks were maintained at the 6-month visit, although the magnitude of those differences tended to diminish. This illustrates the long-term effect of the COB-MS intervention in this sample of participants suggesting a potential sustained-over-time benefit for this cohort.

### Progression

The progression criteria set and achievement of same is presented in Table 5. Through the ACCEPT criteria [40], progression was further evaluated via the traffic light system [39].

**Table 5:** Progression Criteria [39, 40]

Criterion	Outcome (Traffic Light System)	Modification or Note
90% of participants will complete the intervention	Green (Go)	Intervention was moved online due to the impacts of the COVID-19 pandemic
The rate of unblinding will be 50% or lower	Green (Go)	Data was collected online which may have inadvertently reduced the chance of accidental unblinding- e.g. research staff seeing the COB-MS handbook in the participant’s house
70% of participants will report benefits from the intervention and would recommend it to others	Green (Go)	Qualitative data collected (see 44)
Participant recruitment complete in six months	Green (Go)	Completed in four months
Feasibility and appropriateness of the trial design	Amber (Amend)	Trial design was feasible and appropriate. A change will be introduced for a definitive trial based on participant feedback and feasibility results-the inclusion of an in-person and online arm for COB-MS delivery
Feasibility and appropriateness of the mechanics, management and safety of interventions	Green (Go)	No further changes. No adverse events reported
Acceptability and efficiency of implementing the research procedures	Green (Go)	All future participant data will be collected remotely

The appropriateness of progression to a full trial was established. Feasibility and appropriateness of the trial design was confirmed, as well as management and safety of interventions and acceptability and efficiency of implementing the research procedures. These have been confirmed by the TSC.

## Discussion

The COB-MS intervention and trial procedures were found to be feasible. The intervention was well-accepted by both participants with MS and occupational therapists. The progression criteria for the feasibility trial were also met.

Evidence of preliminary efficacy of the COB-MS intervention was found when comparing the COB-MS group to the wait-list control group on daily life function through the primary outcome of goal attainment. It is reasonable to suggest that this positive effect may have resulted from the dual support received by participants from not only the occupational therapist, but also other participants engaged in group sessions – which may have otherwise not been received (e.g. due to friends and family’s lack of training or knowledge – be it in occupational therapy; goal setting and attainment; or MS, itself). That is, having the opportunity to engage an occupational therapist who can appropriately support the participant in their goal setting and attainment, alongside the support of other people with the lived experience of MS and understanding its associated challenges – through COB-MS – may have been a defining feature in how participants navigated the intervention and applied it in their daily lives. Notably, the GAS offers a standardized approach to measure the participant’s ability to set and achieve their goals within an acceptable timeframe and has been validated in an MS population [28]. This approach to measurement focuses on the outcome of these goals, rather than their underlying cognitive mechanisms, as outlined in other frameworks that feature goal attainment (e.g. [45]). This is an important consideration, given that such underlying cognitive mechanisms like memory and attentional function as measured by the EMQ-R [34], the BVMT-R [46], and CVLT-II [47], did not reach a significant level of improvement. Neither were cognitive flexibility nor processing speed, as measured by TMT and SDMT respectively [48–50], significantly improved. In light of this analysis of preliminary efficacy, COB-MS does not support goal attainment by targeting the underlying cognitive mechanisms of higher-level executive functions. Rather, goal attainment improved without targeting these foundational domains. This is consistent with related research where GAS was successfully used in cognitive rehabilitation in MS up to seven months and success was not predicted by neurological disability, depression, executive function, or general cognitive ability [51]. Indeed, one of the benefits of using the GAS in neuropsychological rehabilitation in MS is that it can focus in on changes that are important to the person that are often overlooked by standardised measures [29].

Moreover, it is also worth considering that given the negative effects associated with COVID-19 (i.e. stress, fear, uncertainty, as well as isolation and lack of travel due to lockdowns) on mental well-being and cognitive maintenance (add reference) during which the trial took place, it is possible that such adverse effects had impacted participants above and beyond any benefit of the intervention on the cognitive mechanisms assessed. That is, it is possible that the intervention was of benefit to these cognitive mechanisms, but the scores decreased because of impacts created as a result of COVID-19. It is also possible that the null effects on cognitive performance is not a matter of the intervention’s efficacy, rather an artefact of the statistical analysis with respect to the trial’s sample size. Again, given the trial’s focus on feasibility, statistical efficacy was a secondary aim and the sample size reflects this. In the definitive trial, power analysis recommends a sample of xx participants. Results from this larger sample will provide a better indicator of COB-MS’s potentially efficacious effects on cognitive processing.

Fatigue, as measured by the MFIS was the only secondary outcome on which a significant effect of the intervention was yielded, with respect to total score, as well as all subscale scores. This finding reflects the positive qualitative reports by participants regarding the fatigue-management elements of COB-MS, as published elsewhere [44]. It is also possible that, through fatigue management, participants were able to focus more attention on their goals, with fatigue as a potential barrier, reduced. Of note, the MFIS includes a cognitive subscale which (along with other sub-scales) indicated significantly less impact of (cognitive) fatigue on those who were in the COB-MS arm.

Notably, goal attainment activities have previously been associated with fatigue reductions in an MS population [52]. Using fMRI-based goal attainment activities [52], it was observed a decrease in fatigue in the participants who were offered a reward for a task, compared to those who were not offered a reward for doing the same task. The authors [52] also found that there was greater activation in the ventral striatum, a section of the frontal striatal circuits involved in motivation, goal attainment and fatigue [53, 54], in participants who had a monetary goal to attain, in comparison to participants who did not. It is argued that the stimulation of the ventral striatum, through goal attainment activities resulted in reduced fatigue for both MS participants and healthy controls [52]. Given the significant emphasis on goal attainment activities within COB-MS, it is possible that fatigue reduction in the intervention-arm resulted from engagement in goal attainment and motivational activities, relative to controls.

It is also important to note that the beneficial effects of the intervention on the outcomes with significant effects at post-intervention assessment were still present at six-month follow-up. This finding indicates a potential sustained-over-time benefit. It is again acknowledged that the strength of this preliminary efficacy evidence is restricted by the sample size, given the current trial’s focus on feasibility over efficacy.

With regards to feasibility, it is also important to note the context in which this trial took place. The COVID-19 pandemic may have had an impact on participant adherence and retention. Lockdowns may have facilitated greater engagement, because people could not leave their homes, thus potentially improving retention and adherence rates. On the other hand, heightened health anxiety or low mood resulting from isolation may have negatively impacted performance during cognitive testing sessions. People with MS experienced high levels of stress and isolation during the pandemic [55], which can negatively impact executive function [56]. Thus, it is possible that potential cognitive gains arising from the COB-MS programme may have been dampened or counteracted by the stress of the pandemic. A further large-scale definitive trial could investigate the effects of COB-MS on cognitive functioning when participants are not impacted by heightened worry or lockdowns.

### Limitations

The trial was impacted significantly by the COVID-19 pandemic. Practically, such impact meant that many key elements of the trial design required amendment; particularly, data collection and intervention delivery methods. Though data collection methods were found to be requisitely equivalent [25], there are limitations to assessing participants remotely; and although procedures were in place to minimise risks, data were lost to follow-up. For future remote testing, computerised cognitive assessments could be used to prevent any potential information loss which can occur due to poor video call connection. It is also acknowledged that delivering the COB-MS intervention remotely may have excluded certain groups from being able to participate (those without computer or internet access, those without the requisite computer skills to access the intervention online, etc.) but may have also facilitated the involvement of others [44]. This may have limited the diversity of the participants and may have health inequality consequences. Given the change to online delivery of the COB-MS, the potential impact and feasibility of in-person delivery of the intervention remains uncertain; hence, the addition of an online/in-person trial-arm manipulation in future research of COB-MS through a definitive trial.

### Generalisability

The results of the feasibility trial are directly generalisable to a future definitive trial of the COB-MS. Given the trial procedures in place, the study has strong external validity, and the results can be reasonably generalised to an Irish MS cohort living in the community. Further investigation of in-person delivery is needed to establish feasibility. Amendments for future trial include the addition of an in-person arm, the addition of another primary outcome (specifically, the Multiple Sclerosis Impact Scale- 29; [57]) based on qualitative feedback from occupational therapists and people with MS- 44), and the addition of both a mixed-methods process evaluation to assist in the implementation of the COB-MS and cost-effectiveness analysis to help inform healthcare decision makers on whether to allocate resource to COB-MS.

## Conclusion

The COB-MS is acceptable and has strong potential to be a clinically useful intervention to address the cognitive symptoms seen in multiple sclerosis. The results from the current research provide a strong basis for a pathway to a future definitive trial of COB-MS, in light of results suggesting both its feasibility and preliminary, clinical efficacy.

## Electronic supplementary material

Below is the link to the electronic supplementary material.

Supplementary file1 (PDF 480 KB)

## Data Availability

Data for this study are freely available through Open Science Framework (Registration 10.17605/OSF.IO/T2J3Q).

## Appendix 1

Table 6

**Table 6:** CONSORT 2010 checklist of information to include when reporting a pilot or feasibility trial

Section/Topic	Item No	Checklist item	Reported on page
Title and abstract			
	1a	Identification as a pilot or feasibility randomised trial in the title	1
	1b	Structured summary of pilot trial design, methods, results, and conclusions (for specific guidance see CONSORT abstract extension for pilot trials)	1
Introduction			
Background and objectives	2a	Scientific background and explanation of rationale for future definitive trial, and reasons for randomised pilot trial	2–3
	2b	Specific objectives or research questions for pilot trial	3
Methods			
Trial design	3a	Description of pilot trial design (such as parallel, factorial) including allocation ratio	3–4
	3b	Important changes to methods after pilot trial commencement (such as eligibility criteria), with reasons	4
Participants	4a	Eligibility criteria for participants	5
	4b	Settings and locations where the data were collected	4 and 7
	4c	How participants were identified and consented	4–5
Interventions	5	The interventions for each group with sufficient details to allow replication, including how and when they were actually administered	36–38
Outcomes	6a	Completely defined prespecified assessments or measurements to address each pilot trial objective specified in 2b, including how and when they were assessed	7
	6b	Any changes to pilot trial assessments or measurements after the pilot trial commenced, with reasons	n/a
	6c	If applicable, prespecified criteria used to judge whether, or how, to proceed with future definitive trial	7
Sample size	7a	Rationale for numbers in the pilot trial	8
	7b	When applicable, explanation of any interim analyses and stopping guidelines	n/a
Randomisation:			
Sequence generation	8a	Method used to generate the random allocation sequence	8
	8b	Type of randomisation(s); details of any restriction (such as blocking and block size)	8
Allocation concealment mechanism	9	Mechanism used to implement the random allocation sequence (such as sequentially numbered containers), describing any steps taken to conceal the sequence until interventions were assigned	8
Implementation	10	Who generated the random allocation sequence, who enrolled participants, and who assigned participants to interventions	8
Blinding	11a	If done, who was blinded after assignment to interventions (for example, participants, care providers, those assessing outcomes) and how	9
	11b	If relevant, description of the similarity of interventions	n/a
Statistical methods	12	Methods used to address each pilot trial objective whether qualitative or quantitative	9–10
Results			
Participant flow (a diagram is strongly recommended)	13a	For each group, the numbers of participants who were approached and/or assessed for eligibility, randomly assigned, received intended treatment, and were assessed for each objective	11
	13b	For each group, losses and exclusions after randomisation, together with reasons	10–11
Recruitment	14a	Dates defining the periods of recruitment and follow-up	10
	14b	Why the pilot trial ended or was stopped	10
Baseline data	15	A table showing baseline demographic and clinical characteristics for each group	12–13
Numbers analysed	16	For each objective, number of participants (denominator) included in each analysis. If relevant, these numbers should be by randomised group	16–17
Outcomes and estimation	17	For each objective, results including expressions of uncertainty (such as 95% confidence interval) for any estimates. If relevant, these results should be by randomised group	16–17
Ancillary analyses	18	Results of any other analyses performed that could be used to inform the future definitive trial	14–17 and online material
Harms	19	All important harms or unintended effects in each group (for specific guidance see CONSORT for harms)	18
	19a	If relevant, other important unintended consequences	n/a
Discussion			
Limitations	20	Pilot trial limitations, addressing sources of potential bias and remaining uncertainty about feasibility	22
Generalisability	21	Generalisability (applicability) of pilot trial methods and findings to future definitive trial and other studies	22
Interpretation	22	Interpretation consistent with pilot trial objectives and findings, balancing potential benefits and harms, and considering other relevant evidence	19–22
	22a	Implications for progression from pilot to future definitive trial, including any proposed amendments	18–19; 22
Other information			
Registration	23	Registration number for pilot trial and name of trial registry	23
Protocol	24	Where the pilot trial protocol can be accessed, if available	24; 27
Funding	25	Sources of funding and other support (such as supply of drugs), role of funders	23
	26	Ethical approval or approval by research review committee, confirmed with reference number	23

## Appendix 2

Table 7

**Table 7:** The Template for Intervention Description and Replication (TIDieR; Hoffman et al., 2014) checklist

	Item	Description
1	Phrase that describes the intervention	An occupational therapy intervention aimed at improving daily life functioning for people with multiple sclerosis who are experiencing cognitive difficulties
2	Rationale, theory, or goal of the elements essential to the intervention	The COB-MS is a patient-centred holistic programme which consists of eight sessions- two individual and six group-based. It focuses on managing demands of employment and daily life through education, remediation and adaption using compensatory strategies, routines and learning new techniques that can be integrated into daily contexts. It recognises the impact of emotion, motivation and other non-cognitive functions The COB-MS looks at the context/environment within which the person lives in order to make strategies meaningful to the participant. The programme aims to help people meet their goals while managing their cognitive challenges. There is an emphasis on group discussion and peer learning. Participants have a chance to practice strategies in the group and at home. All sessions took place online (individual and group) due to impact of COVID-19 The aim, through the COB-MS, is to equip people with MS with strategies to manage their own symptoms. A number of steps have been taken in designing the COB-MS which addressed the maintenance of gains and continuation of strategies developed through the intervention – these include incorporating behaviour change principles into the COB-MS, integrating home activities into weekly routines, providing people with MS with handbooks which detail each COB-MS session, setting regular goals and having a definitive list of strategies that work for that person at the end of the last COB-MS session
3	Materials provided	The occupational therapist is provided with a facilitator handbook and participants with MS provided with a participant handbook that details the content of the intervention. The handbooks are the essential components of the intervention. The handbooks summarise each session and have space for in-group and homework activities. A customised version is also available for individuals who require a larger font. Participants can keep the handbooks at the end of the intervention period and are encouraged to review them regularly. As the COB-MS is still subject to research and development, the handbooks are not yet available publicly
4	Describe each of the procedures, activities, setting and/or processes used in the intervention	The COB-MS was initially planned to take place in a community setting but moved online due to impact of COVID-19. The COB-MS group received two hours of one-to-one occupational therapy as well as six-hours of group-based occupational therapy focused on cognitive rehabilitation. Each group session has a mixture of theory/background discussion on aspects of cognition (10 min) and strategies (15 min). This is followed by opportunities to practice strategies (15 min) and discuss usefulness, application to own life and goals (20 min). The focus of COB-MS is on translation to daily life tasks
5	Who is intervention provider?	COB-MS is designed to be run by qualified occupational therapists with experience and expertise in managing everyday life challenges. The occupational therapists received training in COB-MS, as well as ongoing support, if required. The lead applicant (SH; author of handbook) was responsible for training the occupational therapists. By using multiple therapists to carry out the intervention, feasibility of the COB-MS in practice was evaluated
6	Describe the modes of delivery	Session one involves an initial online meeting with an occupational therapist who introduces the programme and helps the participant set some personal goals. There are then six once-weekly group sessions with a small group (recommend 5–6 people for online delivery). The group sessions are followed by a final individual session that takes place two weeks after the last group session. Each COB-MS session is set to last between 60–90 min. Clinical judgement is required to assess the group/individual in terms of tolerance and fatigue
7	Location(s) where the intervention occurs	All sessions (individual and group) took place online. Both the participant and the occupational therapist ensured that they have privacy for the session. This was especially important for the participants during the group sessions
8	When and How Much?	There are eight COB-MS sessions which run over nine weeks. They are once-weekly with the final session happening two weeks after the penultimate session. The COB-MS sessions last between 60–90 min. The recommended duration spent on homework is 30 min per day 5 days/week, but this is likely to vary. Each participant received the intervention once
9	Tailoring	The intervention is planned to be personalised, through the setting of individual goals in the first session. Participants are encouraged to apply what they are learning in the group sessions to their own lives and goals. Participants can also personalise the intervention when they have one-to-one sessions with the occupational therapist. The intervention dose and content are the same for all participants
10	Modifications	The intervention was not modified during the course of the study. Fidelity measures were in place to address this
11	Planned fidelity assessment	Occupational therapists kept a record of the intervention session content, length, and other important information after each session. A sample (two per COB-MS group) of COB-MS sessions per occupational therapist was audio-recorded and compared for intervention fidelity, with permission from occupational therapist and group members
12	The extent to which the intervention was delivered as planned	This data was captured in the study to allow for future trial planning. Participants were asked to rate the occupational therapists at the end of the COB-MS sessions on several areas relevant to the delivery of the intervention. This was anonymous, data was pooled, and occupational therapists were aware of this in advance

## Declarations

### Protocol

The protocol for this trial was published: Dwyer, C. P., Alvarez-Iglesias, A., Joyce, R., Counihan, T. J., Casey, D., & Hynes, S. M. (2020). Evaluating the feasibility and preliminary efficacy of a Cognitive Occupation-Based programme for people with Multiple Sclerosis (COB-MS): protocol for a feasibility cluster-randomised controlled trial. *Trials*, *21*(1), 1–12.] and the protocol was subsequently updated in light of COVID-19 [Dwyer, C.P., Alvarez-Iglesias, A., Joyce, R. et al. (2023). Evaluating the feasibility and preliminary efficacy of a Cognitive Occupation-Based programme for people with Multiple Sclerosis (COB-MS): an update to the protocol for a feasibility cluster-randomised controlled trial. Trials 24, 48. 10.1186/s13063-023–07080-y].
