# Supplementary material for: A cluster-randomised controlled feasibility trial evaluating the Cognitive Occupation-Based programme for people with Multiple Sclerosis (COB-MS)
Source: Neurol Sci. 2024 Sep 24;46(1):445–62. doi: 10.1007/s10072-024-07757-5 (PMC11698819; doi:10.1007/s10072-024-07757-5)

Title: A cluster-randomised controlled feasibility trial evaluating the Cognitive Occupation-Based programme for people with Multiple Sclerosis (COB-MS)

Journal name: Journal of Neurology

Author names: Sinéad M. Hynes*, Christopher P. Dwyer, Alberto Alvarez-Iglesias, Fionnuala Rogers, Robert Joyce, Megan Oglesby, Anusha Moses, Eimear Bane, Timothy J. Counihan, Beatrice Charamba, and COB-MS PPI Advisory Panel.

Affiliation and e-mail address of the corresponding author: Discipline of Occupational Therapy, School of Health Sciences, College of Medicine, Nursing and Health Sciences, University of Galway, Galway, Ireland.

***Corresponding author**

E-mail: sinead.hynes@universityofgalway.ie

Supplementary Material: Secondary Outcome Measures for COB-MS – Collated Figures

Figure 1: Symbol Digit Modality Test and Generalised Self-Efficacy Scale

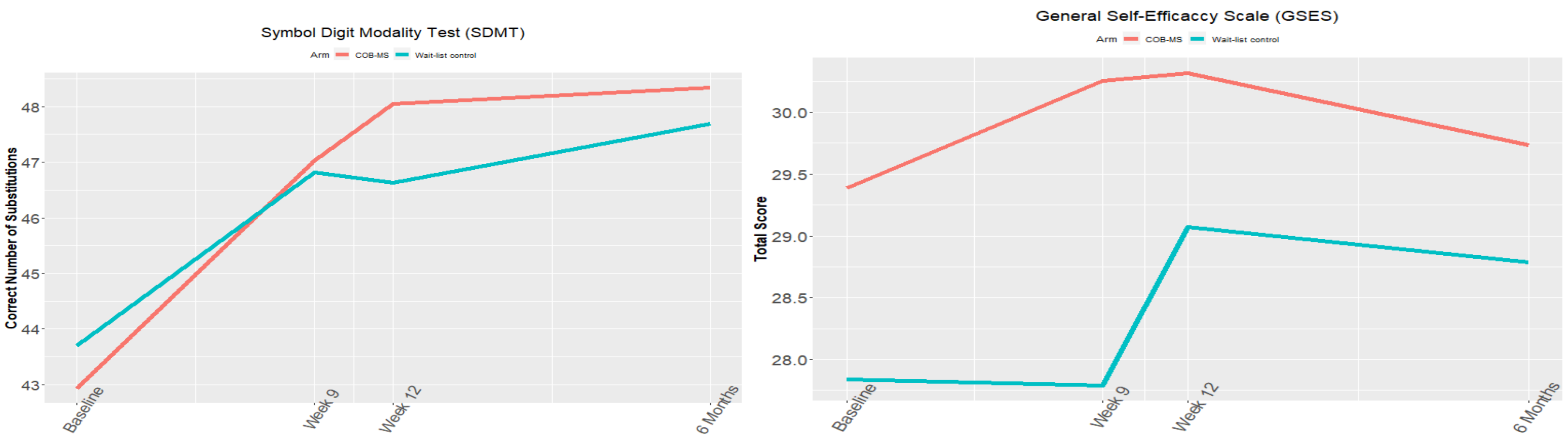

Figure 2 Brief Visuospatial Memory Test-Revised

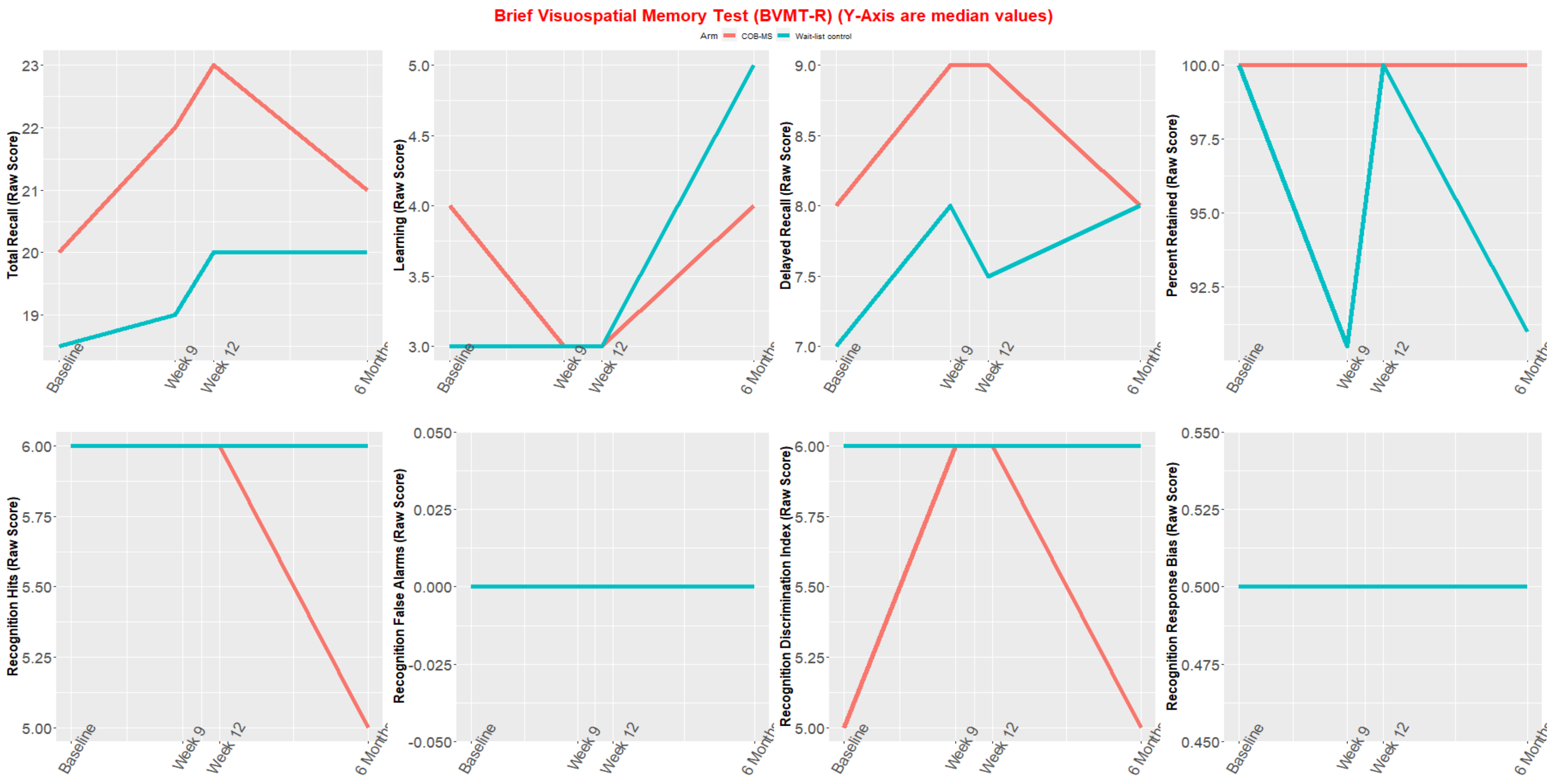

Figure 3 California Verbal Learning Test II

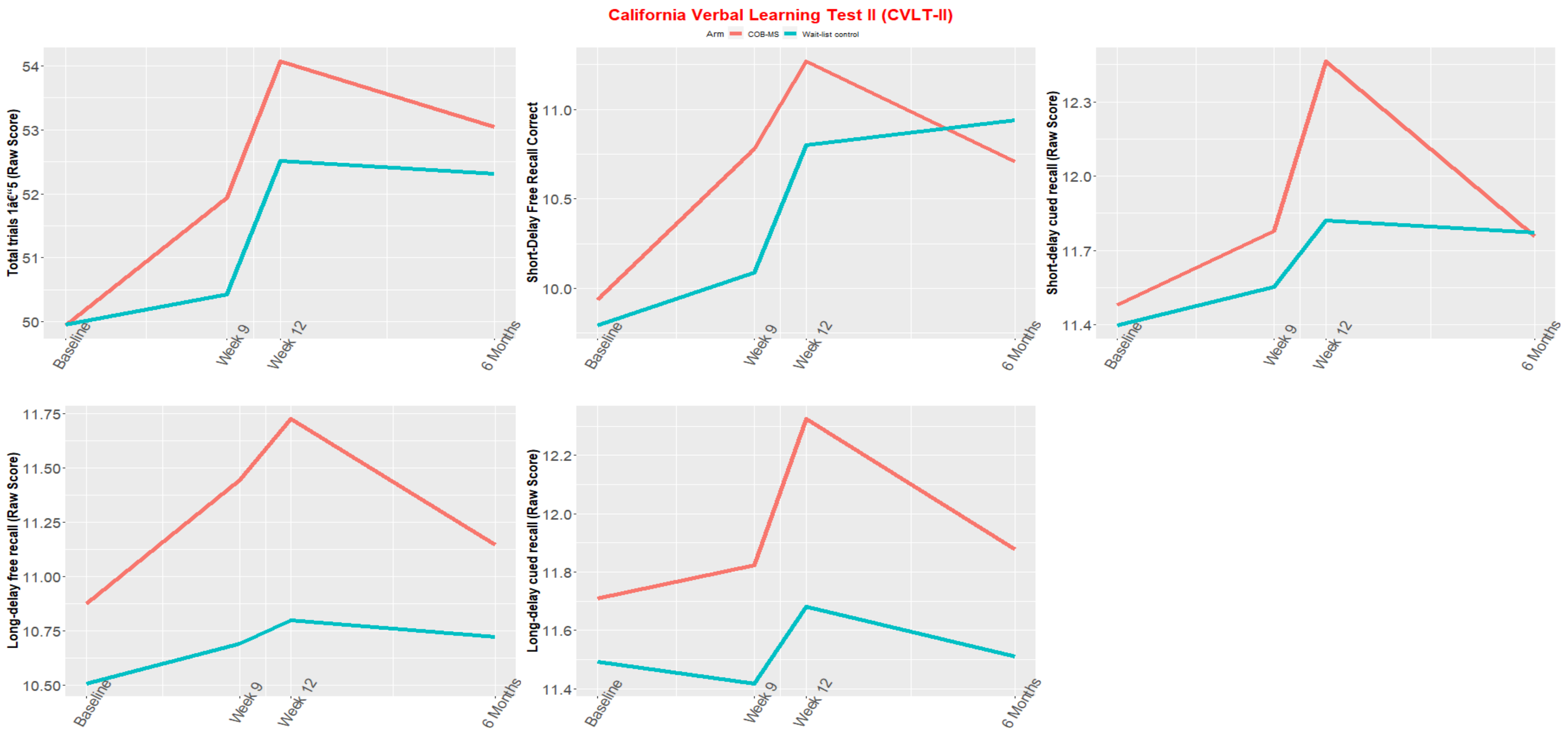

Figure 4 Trail Making Test A and B

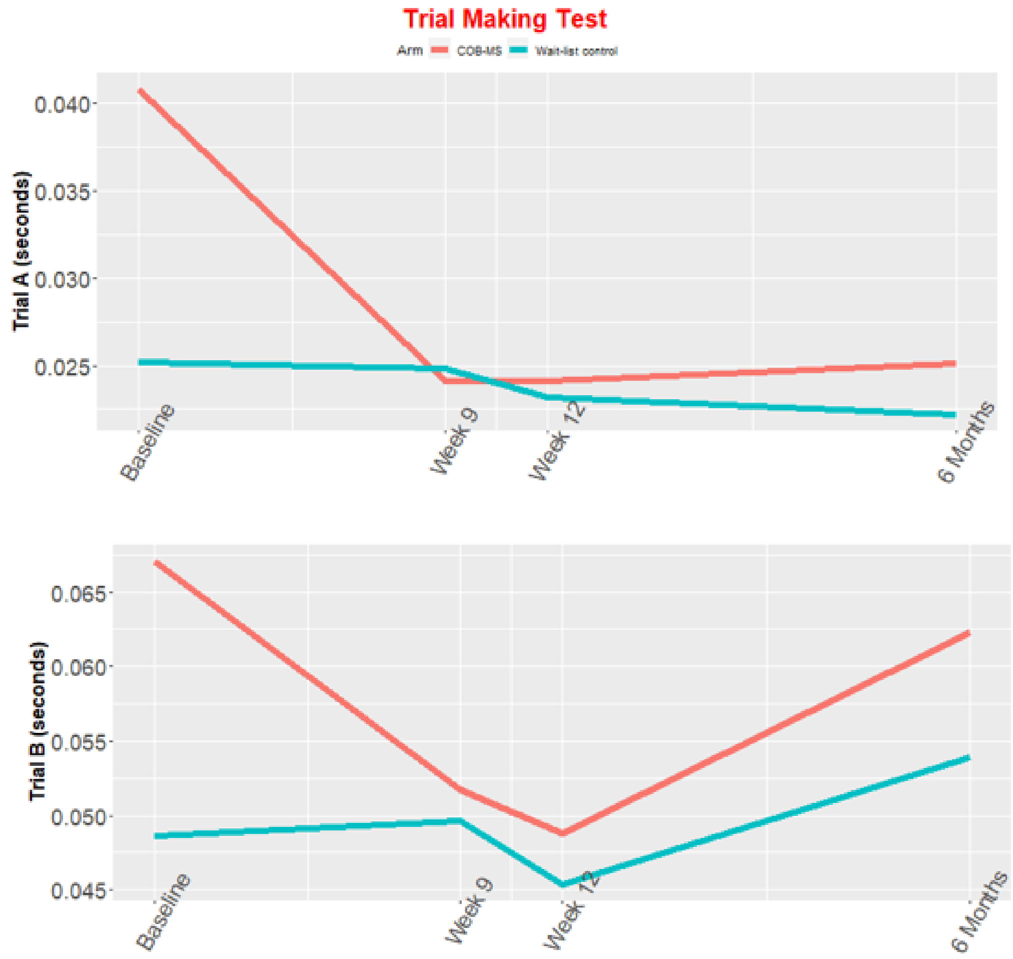

Figure 5 Everyday Memory Questionnaire Revised and General Health Questionnaire

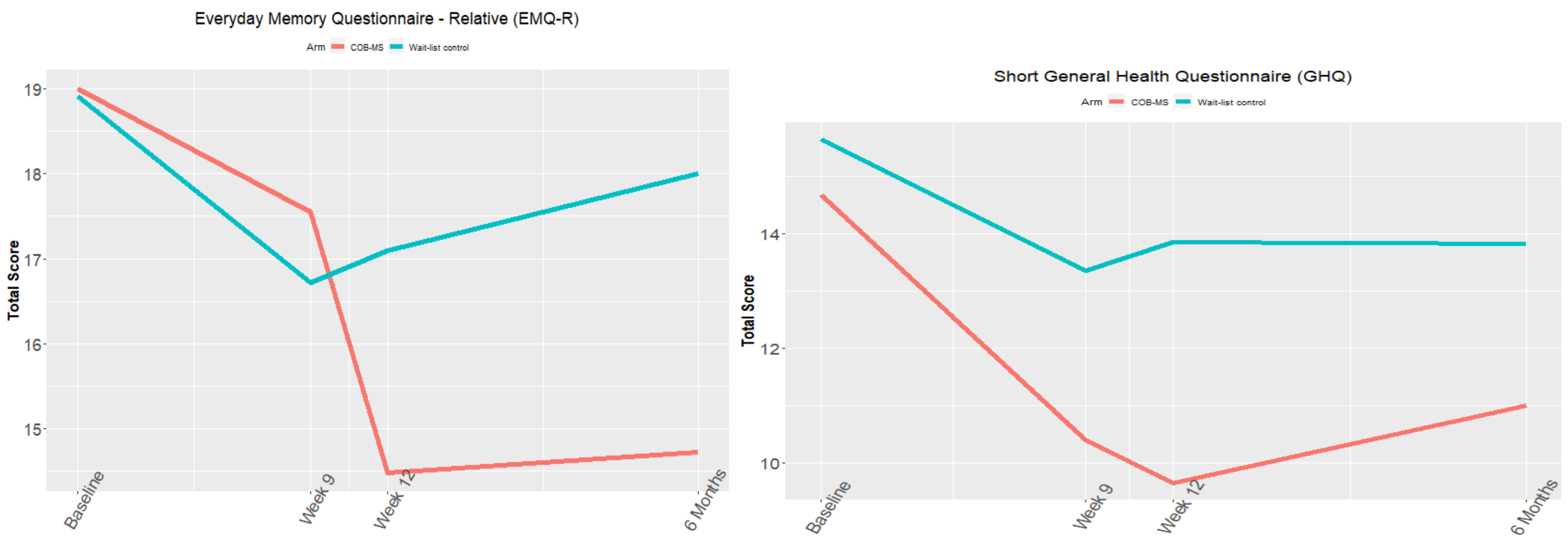

Figure 6 Modified Fatigue Impact Scale

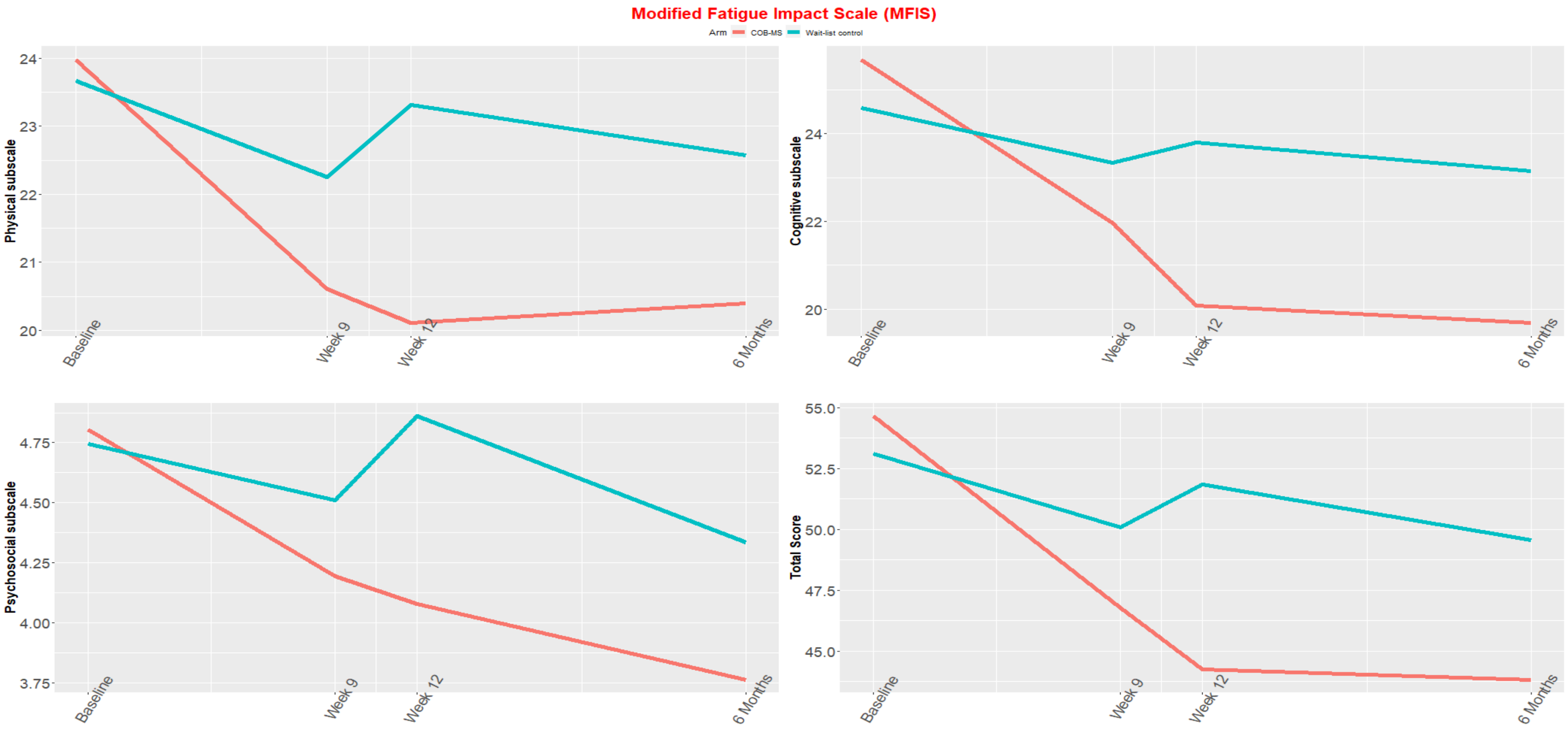

Figure 7 Multiple Sclerosis Quality of Life -54

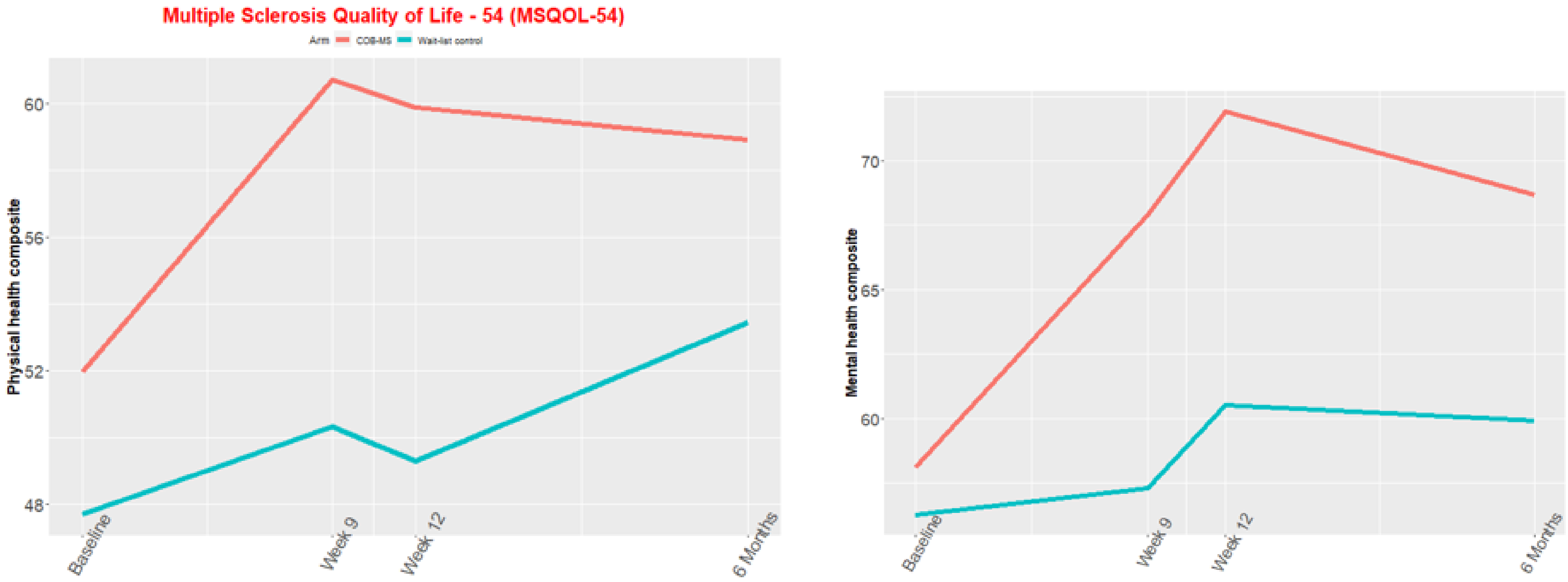

Supplement: Supplementary file 1 — Supplementary file1 (PDF 480 KB) [file 10072_2024_7757_MOESM1_ESM.pdf]
